# Dual Strands of Pre-*miR-149* Inhibit Cancer Cell Migration and Invasion through Targeting *FOXM1* in Renal Cell Carcinoma

**DOI:** 10.3390/ijms18091969

**Published:** 2017-09-13

**Authors:** Atsushi Okato, Takayuki Arai, Yasutaka Yamada, Sho Sugawara, Keiichi Koshizuka, Lisa Fujimura, Akira Kurozumi, Mayuko Kato, Satoko Kojima, Yukio Naya, Tomohiko Ichikawa, Naohiko Seki

**Affiliations:** 1 Department of Functional Genomics, Chiba University Graduate School of Medicine, 2608670 Chiba, Japan; aexa5919@chiba-u.jp (A.O.); agda0043@chiba-u.jp (T.A.); yasutaka1205@chiba-u.jp (Y.Y.); afha7393@chiba-u.jp (S.S.); aeha4003@chiba-u.jp (K.K.); adfa2718@chiba-u.jp (A.K.); mayukokato@chiba-u.jp (M.K.); 2 Department of Urology, Chiba University Graduate School of Medicine, 2608670 Chiba, Japan; tomohiko_ichikawa@faculty.chiba-u.jp; 3 Department of Biomedical Science, Chiba University Graduate School of Medicine, 2608670 Chiba, Japan; lisaf1227@faculty.chiba-u.jp; 4 Department of Urology, Teikyo University Chiba Medical Center, 2990111 Ichihara, Japan; kojima-s@med.teikyo-u.ac.jp (S.K.); nayay@med.teikyo-u.ac.jp (Y.N.)

**Keywords:** microRNA, *miR-149-5p*, *miR-149-3p*, *FOXM1*, clear cell renal cell carcinoma, antitumor

## Abstract

Our recent studies revealed that dual strands of certain pre-microRNAs, e.g., pre-*miR-144*, pre-*miR-145*, and pre-*miR-150*, act as antitumor microRNAs (miRNAs) in several cancers. The involvement of passenger strands of miRNAs in cancer pathogenesis is a novel concept in miRNA research. The analysis of a miRNA expression signature in clear cell renal cell carcinoma (ccRCC) has revealed that the guide strand of pre-*miR-149* is significantly downregulated in cancer tissues. The aims of this study were to investigate the functional significance of *miR-149*’s guide strand (*miR-149-5p*) and passenger strand (*miR-149-3p*), and to identify the oncogenic genes regulated by these miRNAs in ccRCC cells. The ectopic expression of these miRNAs significantly inhibited cancer cell migration and invasion in ccRCC cells. Forkhead box protein M1 (*FOXM1*) was directly regulated by *miR-149-5p* and *miR-149-3p* in ccRCC cells. Knockdown studies using si-*FOXM1* showed that the expression of *FOXM1* enhanced RCC cell aggressiveness. Interestingly, the analysis of a large number of patients in the The Cancer Genome Atlas (TCGA) database (*n* = 260) demonstrated that patients with high *FOXM1* expression had significantly shorter survival than did those with low *FOXM1* expression (*p* = 1.5 × 10^−6^). Taken together, dual strands of pre-*miR-149* (*miR-149-5p* and *miR-149-3p*) acted as antitumor miRNAs through the targeting of *FOXM1* in ccRCC cells.

## 1. Introduction

Renal cell carcinoma (RCC) is the most common neoplasm of the kidney, and approximately 70–80% of RCCs are classified as clear cell-type (ccRCC) [1]. More than 270,000 patients are diagnosed with RCC every year, which is estimated to constitute approximately 3% of adult malignancies. The incidence and mortality rates of RCC are increasing at a rate of 2–3% per decade [2]. Although surgical resection remains the only known curative treatment for localized RCC, a significant percentage of these patients develop relapses or metastatic disease, which are associated with a poor prognosis [3]. Recently developed targeted molecular therapies and immunomodulatory agents are currently being widely used for patients with metastatic or recurrent RCC [4]. However, those curative approaches are restricted to RCC patients in advanced stages of disease and the 5-year survival rate of the patients is only 5–10% [5]. Therefore, to improve outcomes in patients with RCC, it is necessary to fully elucidate the molecular mechanisms of metastatic RCC based on new genomic approaches.

MicroRNAs (miRNAs) belong to a class of noncoding RNA molecules that fine-tune the expression of protein coding/noncoding RNAs by repressing translation or cleaving RNA transcripts in a sequence-dependent manner [6]. Overexpression of oncogenic miRNAs and dysfunction of antitumor miRNAs are associated with human cancer pathogenesis [7]. A single miRNA species can regulate the expression of hundreds or thousands of different mRNAs, and an individual species of mRNA can be regulated by multiple different miRNAs in normal cells [8]. Therefore, aberrantly expressed miRNAs can disrupt regulatory miRNA–mRNA networks in cancer cells.

The construction of expression signatures of miRNAs in human cancers is an effective strategy to identify aberrantly expressed miRNAs in cancer cells. We have constructed miRNA signatures in several types of human cancers, including RCC [9,10,11,12]. From analyses of the signatures, we have found that some passenger strands of miRNAs were significantly reduced in cancer tissues, suggesting that passenger strands of miRNAs have an antitumor function in cancer cells, as do guide strands of miRNAs. In miRNA biogenesis, one strand of mature miRNA (the guide strand) is loaded into the miRNA-induced silencing complex (RISC) that targets mRNA degradation and translational repression in processing bodies [13]. In contrast, the passenger strand of miRNA was previously thought to be degraded and to have no function [14,15,16]. Interestingly, our recent studies demonstrated that the dual strands (guide strand and passenger strand) of certain pre-miRNAs, such as pre*-miR-144*, pre-*miR-145*, pre-*miR-139*, and pre-*miR-150*, acted as antitumor miRNAs through the targeting of several oncogenic genes in several cancers [17,18,19]. Novel approaches to the analysis of dual strand miRNA-regulated RNA networks in cancer cells may provide new insights into the pathogenic development of human cancers.

Our original miRNA signature of RCC led us to focus on the dual strands of pre-*miR-149*, i.e., *miR-149-5p* (the guide strand) and *miR-149-3p* (the passenger strand) [9]. The aims of this study were to investigate the functional significance of these miRNAs and to identify coordinately regulated oncogenic genes in RCC cells. The identification of the function of passenger strands of miRNAs and novel mechanisms of miRNA-mediated gene regulation enhance our understanding of the molecular pathways underlying RCC initiation, development, and metastasis.

## 2. Results

### 2.1. Expression Levels of miR-149-5p and miR-149-3p in ccRCC Specimens and Cell Lines

**Figure 1:**
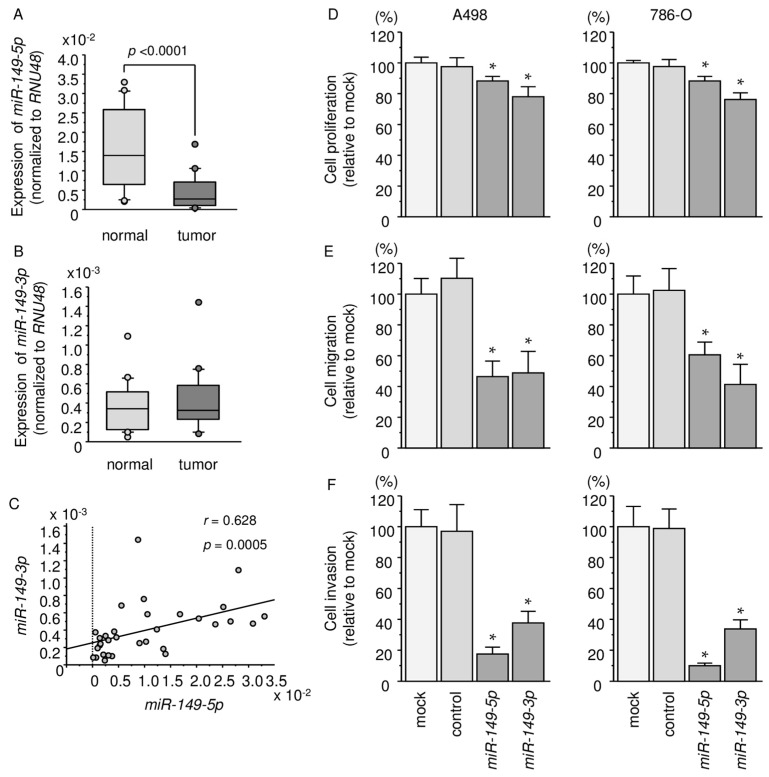
Analysis of *miR-149-5p* and *miR-149-3p* expression in clinical specimens and functional assays in renal cell carcinoma (RCC) cell lines (A498 and 786-O cells) following *miR-149-5p* and *miR-149-3p* transfection. (**A**,**B**) Expression levels of *miR-149-5p* and *miR-149-3p* in RCC clinical specimens were determined by qRT-PCR. Data were normalized to *RNU48* expression; (**C**) Correlation between the relative expression levels of *miR-149-5p* and *miR-149-3p*; (**D**) Cell proliferation was determined by XTT assays. * *p* < 0.0001; (**E**) Cell migration activity was determined by wound-healing assays. * *p* < 0.0001; (**F**) Cell invasion activity was determined using Matrigel invasion assays. * *p* < 0.0001.

We evaluated the expression levels of *miR-149-5p* and *miR-149-3p* in kidney tissues (cancerous specimens and adjacent non-cancerous specimens). The patients’ backgrounds are summarized in Table 1. The expression levels of *miR-149-5p* were significantly lower in cancerous tissues than in normal tissues, but there were no significant differences in the expression levels of *miR-149-3p* between cancerous tissues and non-cancerous tissues (*miR-149-5p*: *p* < 0.0001, *miR-149-3p*: *p* = 0.473; Figure 1A,B). Comparing the expression levels of two miRNAs, *miR-149-5p* (guide strand) and *miR-149-3p* (passenger strand) in RCC cells, our data showed that the expression level of the *miR-149-5p* strand is more abundant than that of the *miR-149-3p* strand in RCC cells (Figure 1A,B). Spearman’s rank tests showed positive correlations between the expression level of *miR-149-5p* and *miR-149-3p* (*r* = 0.628 and *p* = 0.0005; Figure 1C).

### 2.2. Effects of Ectopic Expression of miR-149-5p and miR-149-3p on Cell Proliferation, Migration, and Invasion Assays in RCC Cell Lines

To examine the functional roles of *miR-149-5p* and *miR-149-3p*, we performed gain-of-function studies by using A498 and 786-O cells transfected with mature miRNAs.

XTT assays revealed that proliferation was significantly inhibited in A498 and 786-O cells transfected with *miR-149-5p* and *miR-149-3p* in comparison with mock or miR-control-transfected cells (*p* < 0.0001; Figure 1D). Wound-healing and Matrigel invasion assays demonstrated significant inhibition of cell migration and invasion in *miR-149-5p* and *miR-149-3p* transfectants (*p* < 0.0001; Figure 1E,F).

### 2.3. Both miR-149-5p and miR-149-3p Bind to Ago2

**Figure 2:**
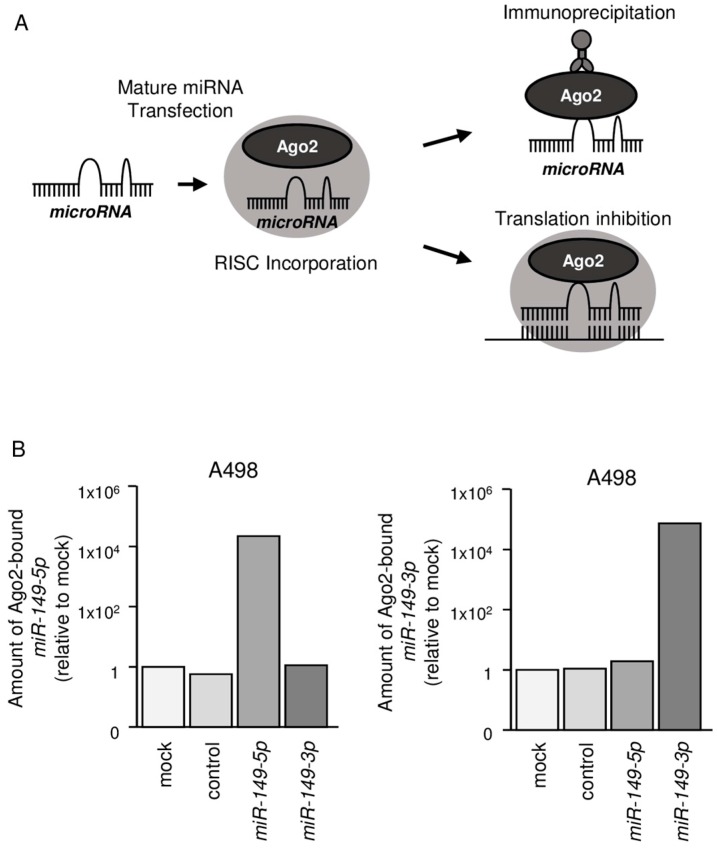
Both *miR-149-5p* and *miR-149-3p* bound to Ago2. (**A**) Schematic illustration of microRNA (miRNA) detection method. Isolation of miRNA-induced silencing complex (RISC) incorporated miRNAs by Ago2 immunoprecipitation; (**B**) Expression levels of *miR-149-5p* and *miR-149-3p* after transfection with *miR-149-5p* or *miR-149-3p*. We used *miR-26a* as an internal control.

We hypothesized that both *miR-149-5p* and *miR-149-3p* may be incorporated into and function as part of the RISC. To test this hypothesis, we performed immunoprecipitation with antibodies targeting Ago2, which plays a central role in the RISC (Figure 2A). After transfection with *miR-149-5p* or *miR-149-3p*, Ago2-bound miRNAs were isolated, and qRT-PCR was carried out to determine whether *miR-149-5p* and *miR-149-3p* were bound to Ago2 (Figure 2B).

After transfection with *miR-149-5p* and immunoprecipitation with anti-Ago2 antibodies, *miR-149-5p* levels were significantly higher than those of mock- or miR control-transfected cells and those of *miR-149-3p*-transfected A498 cells (*p* < 0.0001; Figure 2B). Similarly, after transfection with *miR-149-3p* and immunoprecipitation with anti-Ago2 antibodies, *miR-149-3p* levels were significantly higher than those of mock- or miR control-transfected cells and those of *miR-149-5p*-transfected A498 cells (*p* < 0.0001; Figure 2B).

### 2.4. Screening of Target Genes Regulated by miR-149-5p and miR-149-3p in RCC Cells

**Figure 3:**
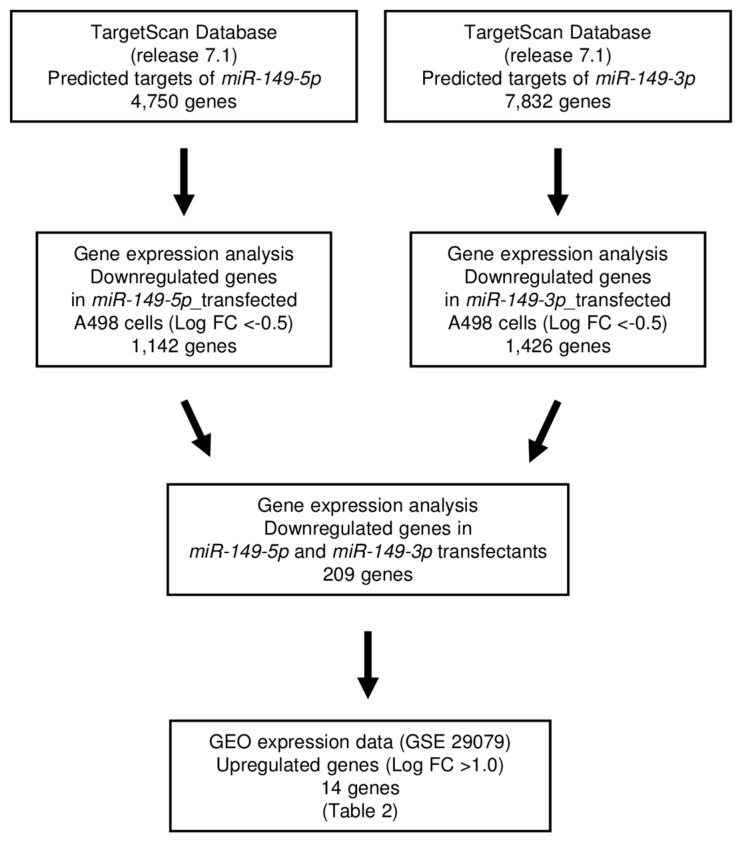
The strategy underlying the analysis of *miR-149-5p* and *miR-149-3p* target genes in A498 cells. FC: fold-change.

Next, we sought to obtain further insights into the molecular mechanisms regulated by antitumor *miR-149-5p* and *miR-149-3p* in RCC cells. Thus, we screened the genes regulated by those miRNAs using in silico and genome-wide gene expression analyses. First, we performed in silico analyses. An analysis of the TargetScan database showed that 4750 genes and 7832 genes had putative target sites for *miR-149-5p* and *miR-149-3p*, respectively, in their 3′-UTRs. Next, we merged the data for the gene expression analysis data in *miR-149-5p* and *miR-149-3p* transfectants (GSE100746). Finally, we found 14 genes that were upregulated (fold-change log2 > 1.0) in cancer tissues by Gene Expression Omnibus (GEO) database analyses (GEO accession number: GSE22541 and GSE36895). We examined the Kaplan–Meier plot for these genes in RCC using the OncoLnc and cBioPortal database [20,21,22]. Our strategy for analysis is shown in Figure 3. The putative target genes regulated by dual strands of *miR-149*, i.e., *miR-149-5p* and *miR-149-3p*, are summarized in Table 2. Among them, we focused on *FOXM1* because it showed the most significant difference in log rank tests using the OncoLnc database.

### 2.5. Analysis of FOXM1 Expression in ccRCC Clinical Specimens by qRT-PCR and Immunohistochemistry

**Figure 4:**
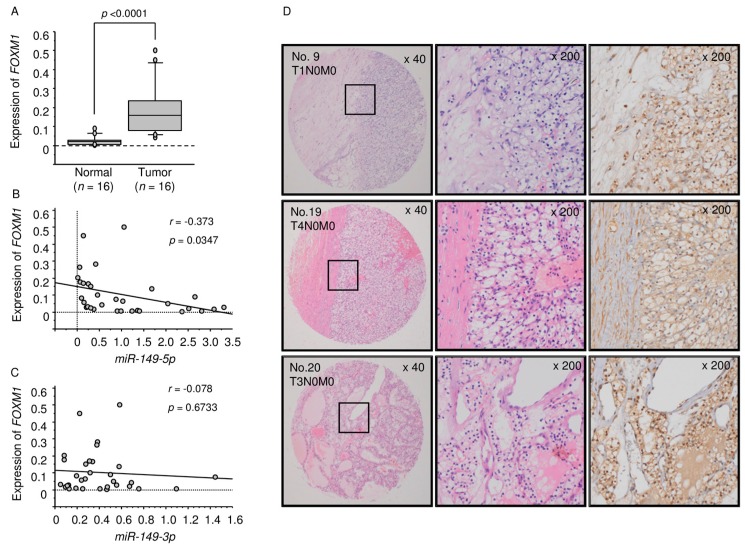
Expression levels of *FOXM1* in ccRCC clinical specimens and TCGA data analysis based on *FOXM1* expression in ccRCC. (**A**) Expression levels of *FOXM1* in ccRCC specimens were significantly upregulated in cancer tissues compared with normal tissues (*p* < 0.0001); (**B**,**C**) The correlation between *FOXM1* and *miR-149-5p*, and *FOXM1* and *miR-149-3p* and (**D**) FOXM1 protein was strongly expressed in several cancer tissues, while low expression was observed in normal tissues using a tissue microarray.

We used qRT-PCR and immunohistochemical staining to examine the expression levels of *FOXM1* in ccRCC specimens. The expression of *FOXM1* was significantly upregulated in cancer tissues compared with normal tissues (*p* < 0.0001; Figure 4A). Spearman’s rank tests showed a negative correlation between the expression levels of *FOXM1* and *miR-149-5p* (*p* = 0.0347 *r* = −0.373; Figure 4B), but there was no correlation between *FOXM1* and *miR-149-3p* (Figure 4C). FOXM1 protein expression was strongly expressed in several cancer tissues (patient No. 9, 19, 20), whereas low expression was observed in normal tissues using a tissue microarray (Figure 4D).

### 2.6. TCGA Database Analysis of Dual Strands of Pre-miR-149 and FOXM1

**Figure 5:**
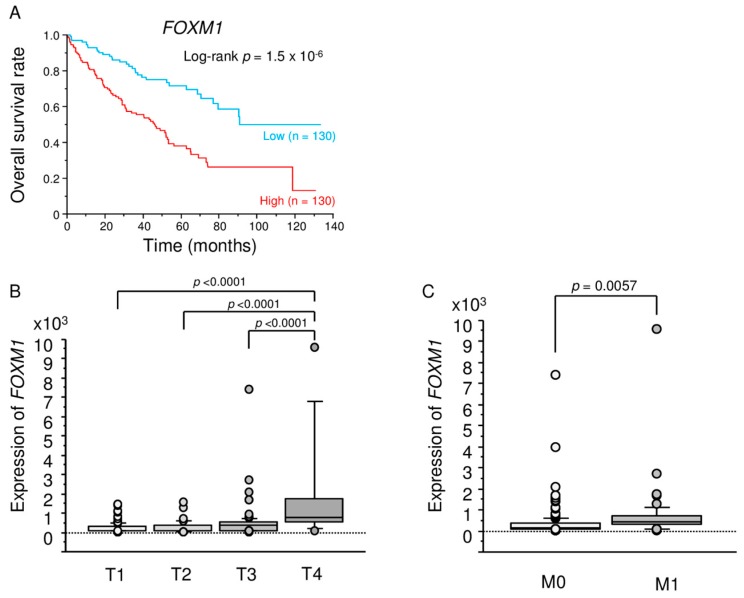
TCGA database analysis of *FOXM1*. (**A**) Kaplan-Meier survival curves for overall survival rates based on *FOXM1* expression in RCC (*p* = 1.5 × 10^−6^) and (**B**,**C**) The relationships between *FOXM1* expression and tumor stage and metastasis in ccRCC.

We analysed Kaplan-Meier overall survival (OS) curves according to the expression levels of dual strands of pre-*miR-149* and *FOXM1* and the relationships between *FOXM1* expression and tumor stage and metastasis in ccRCC using the TCGA-KIRC database. The TCGA dataset showed that high expression of *miR-149-5p* was associated with a poor prognosis of the patients with RCC. The *miR-149-3p* data was not available in this database (data not shown). The Kaplan–Meier curves for OS rates showed that the group with high expression of *FOXM1* had a significantly shorter survival than the low expression group in ccRCC (*p =* 1.5 × 10^−6^, Figure 5A). The expression levels of *FOXM1* were significantly increased in advanced T stage cases and metastatic cases (Figure 5B,C).

### 2.7. Regulation of FOXM1 Expression by miR-149-5p and miR-149-3p in RCC Cells

**Figure 6:**
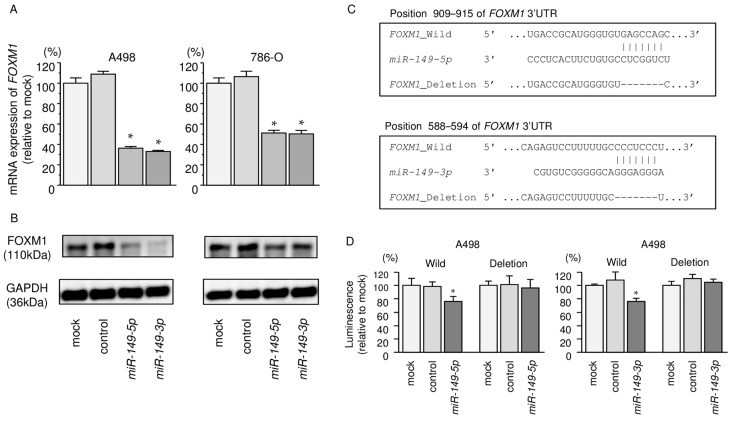
Direct regulation of *FOXM1* by *miR-149-5p and miR-149-3p* in RCC cell lines. (**A**) *FOXM1* mRNA expression in RCC cell lines was evaluated by qRT-PCR. *GAPDH* was used as an internal control. * *p* < 0.0001; (**B**) FOXM1 protein expression in RCC cell lines was evaluated by Western blot analyses. GAPDH was used as a loading control; (**C**)The *miR-149-5p* or *miR-149-3p* binding site in the 3′-UTR of *FOXM1* mRNA; (**D**) Dual Luciferase reporter assays using vectors encoding putative *miR-149-5p* and *miR-149-3p* target sites of the *FOXM1* 3′-UTR (positions 909–915 and 588–594) for both wild-type and deleted regions. Normalized data were calculated as ratios of *Renilla*/*Firefly* luciferase activities. * *p* < 0.001.

Our studies revealed that *FOXM1* mRNA was significantly reduced in both *miR-149-5p* and *miR-149-3p* transfectants in comparison with mock or miR-control transfectants (*p* < 0.0001 and *p* < 0.0001; Figure 6A). The expression of FOXM1 protein was also repressed in these miRNA transfectants (Figure 6B). The target prediction databases indicated that both *miR-149-5p* and *miR-149-3p* had one putative target site in the 3′-UTR of *FOXM1* for *miR-149-5p* (position 909–915) and *miR-149-3p* (positions 588–594) (Figure 6C). We performed a dual luciferase reporter assay. The TargetScan database identified one putative target site in the 3′-UTR of *FOXM1* for *miR-149-5p* (position 909–915) and *miR-149-3p* (positions 588–594). We used vectors encoding a partial wild-type sequence of the 3′-UTR of *FOXM1* mRNA, including the predicted *miR-149-5p* and *miR-149-3p* target site, or a vector lacking the *miR-149-5p* and *miR-149-3p* target sites. We found that the luminescence intensity was significantly reduced by co-transfection with *miR-149-5p* or *miR-149-3p* and the vector carrying the wild-type 3′-UTR of *FOXM1* (*p* < 0.001; Figure 6D).

### 2.8. Effects of Silencing FOXM1 on Cell Proliferation, Migration, and Invasion in RCC Cells

**Figure 7:**
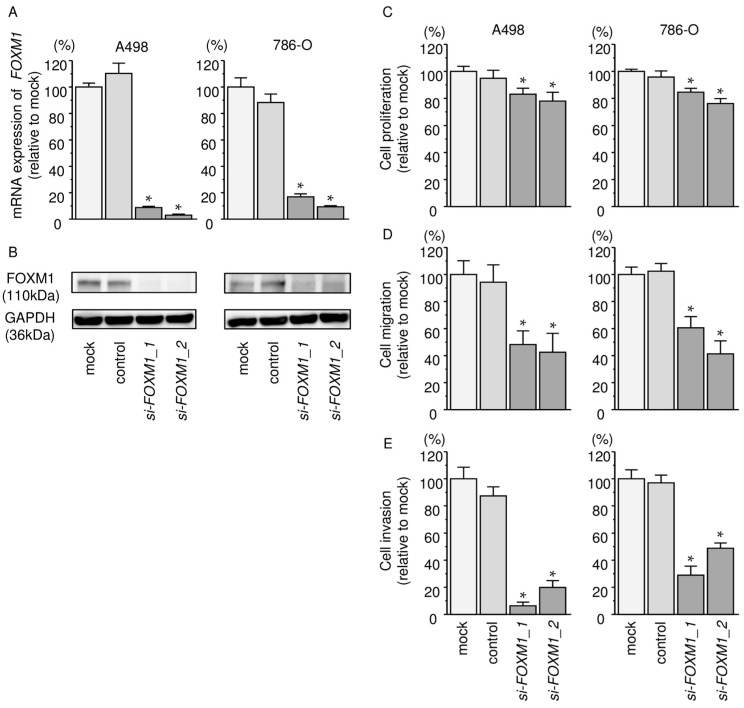
*FOXM1* mRNA and FOXM1 protein expression after si*-FOXM1* transfection and the effects of *FOXM1* silencing in RCC cell lines. (**A**) *FOXM1* mRNA expression in RCC cell lines was evaluated by qRT-PCR. *GAPDH* was used as an internal control; (**B**) FOXM1 protein expression in RCC cell lines was evaluated by Western blot analysis. GAPDH was used as a loading control; (**C**) Cell proliferation was determined using XTT assays, * *p* < 0.0001; (**D**) Cell migration activity was determined by wound-healing assays, * *p* < 0.0001; (**E**) Cell invasion activity was determined using Matrigel invasion assays. * *p* < 0.0001.

We evaluated the knockdown efficiency of *si-FOXM1* transfection in RCC cells. Our present data showed that si-*FOXM1* transfection effectively downregulated *FOXM1* expression in A498 and 786-O cells (Figure 7A,B). Functional assays demonstrated that cell proliferation, migration, and invasion were all inhibited in si*-FOXM1* transfectants compared with mock- or miR control-transfected cells (*p* < 0.0001, Figure 7C–E).

### 2.9. Kaplan–Meier Survival Curves and Genes Affected by miR-149-5p and miR-149-3p

**Figure 8:**
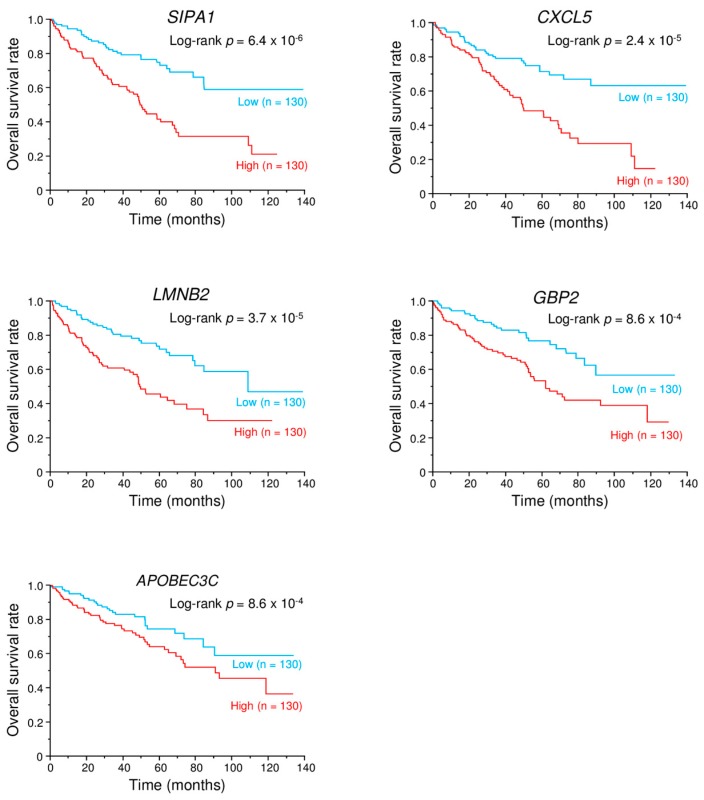
Kaplan-Meier survival analysis. Kaplan-Meier survival curves based on putative target genes regulated by *miR-149-5p* and *miR-149-3p* in patients with ccRCC.

To investigate the contribution of *miR-149-5p* and *miR-149-3p* to the coordinate regulation of genes (Table 2) in ccRCC pathogenesis, we searched the TCGA database. Among 14 genes, the expression of 6 genes (*FOXM1*, *SIPA1*, *CXCL5*, *LMNB2*, *GBP2*, and *APOBEC3C*) was significantly associated with a poor prognosis of patients with ccRCC (Figure 8).

## 3. Discussion

Currently approved targeted therapies have improved the management of patients with metastatic RCC. However, the use of these targeted therapies is restricted to advanced stages of RCC [23]. The elucidation of molecular mechanisms of relapse, metastasis and drug resistance is indispensable to improve the prognosis of RCC patients. To identify novel pathologic molecular mechanisms in RCC, our research group has identified RCC oncogenic pathways based on antitumor miRNAs that were identified by examining RCC miRNA signatures [9]. For example, lysyl oxidase homolog 2 (*LOXL2*) was overexpressed in RCC clinical specimens. Importantly, the antitumor *miR-29* family (*miR-29a/b/c*) targeted *LOXL2* and inhibited cancer cell migration and invasion [24]. More recently, it was found that *miR-101* was significantly reduced in sunitinib-treated RCC tissues and the ectopic expression of *miR-101* inhibited RCC cell aggressiveness through its targeting of ubiquitin-like with PHD and ring finger domains 1 (UHRF1) [9]. Our antitumor miRNAs-based strategy is an attractive approach for the identification of novel regulatory networks in cancer cells.

Our present data demonstrated that dual strands of pre-*miR-149*, *miR-149-5p* and *miR-149-3p*, acted as antitumor miRNAs in RCC cells. The antitumor function of *miR-149-5p* was reported in several other types of cancer [25,26]. In breast cancer, *miR-149-5p* was downregulated by the hyper-methylation of its promoter region and was found to be involved in Adriamycin-resistant breast cancer cells targeting GlcNAc N-deacetylase/N-sulfotransferase-1 (NDST1) [27]. In hepatocellular carcinoma, *miR-149-5p* was significantly downregulated and its expression was correlated with distant metastases and TNM classification [26]. An overexpression of *miR-149-5p* suppressed the migration and invasion activities of hepatocellular carcinoma cells [26]. In colorectal carcinoma, the expression of *miR-149-5p* regulated cell growth, migration, and invasion through its targeting of the EPH receptor B3 gene (*EphB3*), and the knockdown of *EphB3* inhibited tumor growth by in vivo assays [28].

In contrast to *miR-149-5p*, few reports have described the function of *miR-149-3p* in cancer cells. Dioscin is a natural product that induces apoptosis and suppresses tumor growth in pancreatic cells [29]. The expression level of *miR-149-3p* was upregulated by dioscin, and *miR-149-3p* inhibited the Akt1 signaling pathway [29]. A major bioactive component in licorice root is 18β-glycyrrhetinic acid (GRA). It reportedly possesses antitumor effects in several cancers [30,31,32]. In gastric cancer tissues, *miR-149-3p* was downregulated, but its expression was upregulated by GRA treatment [33]. The overexpression of *miR-149-3p* inhibited gastric cancer cell proliferation and cell cycle progression [33]. In these analyses, we did not detect the downregulation of the passenger strand of *miR-149-3p* in RCC tissues. However, the ectopic expression of *miR-149-3p* inhibited cancer cell aggressiveness in RCC cells. This fact indicates that the passenger strand of *miR-149-3p* may possess cancer-suppressing functions in RCC cells. These findings strongly suggest that dual strands of pre-*miR-149* act as antitumor miRNAs in human cancers.

Our recent studies demonstrated that dual strands of certain pre-miRNAs, e.g., pre-*miR-145*, pre-*miR-139*, and pre-*miR-150*, act as antitumor miRNAs. Each guide strand and passenger strand coordinately regulates oncogenic genes as observed in several cancers, e.g., pre-*miR-145*: *MTDH* and *UHRF1*; pre-*miR-139*: *MMP11*; pre-*miR-150*: *SPOCK1* [18,19,34,35]. In this study, we speculate that *miR-149-5p* and *miR-149-3p* work together to regulate pathways in RCC cell progression and metastasis. Our present data showed that a total of 14 genes were putative targets of both *miR-149-5p* and *miR-149-3p* regulation in RCC cells. Interestingly, among the candidate genes, the expression of 6 genes (*FOXM1*, *SIPA1*, *CXCL5*, *LMNB2*, *GBP2*, and *APOBEC3C*) was associated with a poor prognosis of patients with RCC by TCGA analyses (Figure 8).

In this study, we focused on *FOXM1* as a target of the dual strands of pre-*miR-149* and validated the direct binding of these miRNAs to the 3′-UTR using luciferase reporter assays. Past studies have shown that *FOXM1* was regulated by *miR-149-5p* in colorectal cancer and non-small cell lung cancer [36,37]. This is the first study to report that dual strands of pre-*miR-149* coordinately regulate *FOXM1* in RCC cells. FOXM1 is a member of the forkhead transcription family of proteins, and it plays pivotal roles in cell cycle progression in normal cells [38]. An overexpression of *FOXM1* has been detected in a broad range of human cancers, including RCC [39,40,41]. Our present data demonstrated that the knockdown of *FOXM1* in tumor cells by RNAi reduced cell proliferation, migration, and invasion, suggesting that *FOXM1* acts as an oncogene in RCC cells. Moreover, recent meta-analyses have indicated that high expression of *FOXM1* was significantly associated with poor overall survival in most solid tumors [42,43]. Taken together, the expression of *FOXM1* is a useful biomarker for the prognosis of human cancers and a potential therapeutic target in RCC cells. Authors should discuss the results and how they can be interpreted in perspective of previous studies and of the working hypotheses. The findings and their implications should be discussed in the broadest context possible. Future research directions may also be highlighted.

## 4. Materials and Methods

### 4.1. Clinical ccRCC Specimens

Clinical ccRCC specimens were obtained from patients who were admitted to Chiba University Hospital and had undergone radical nephrectomy from 2012 to 2015. A total of 16 pairs of ccRCC specimens and adjacent non-cancerous specimens were collected. The clinicopathological characteristics of the patients are summarized in Table 1. These samples were staged according to the UICC TNM classification [44]. All patients in this study provided written informed consent for tissue donation for research purposes. The protocol was approved by the Institutional Review Boards of Chiba University Hospital (identification code: No. 484, 30 August 2011).

### 4.2. Tissue Collection and Cell Culture

Clinical specimens were immersed in RNAlater (Thermo Fisher Scientific, Waltham, MA, USA) and stored at 4 °C until RNA was extracted. Human ccRCC cells (A498 and 786-O cells) were obtained from the American Type Culture Collection (Manassas, VA, USA).

### 4.3. Quantitative Real-Time Reverse Transcription Polymerase Chain Reaction (qRT-PCR)

Stem-loop RT-PCR (TaqMan MicroRNA Assays; product ID: 002255 for *miR-149-5p* and 002164 for *miR-149-3p*; Applied Biosystems, Foster City, CA, USA) was used for these assays. TaqMan probes and primers for *FOXM1* (product ID: Hs01073586_m1; Applied Biosystems) were assay-on-demand gene expression products. We used *GUSB* (product ID: Hs00939627_m1; Applied Biosystems), *GAPDH* (product ID: Hs02758991_g1; Applied Biosystems), and *RNU48* (product ID: 001006; Applied Biosystems) as internal controls. We first verified the transfection efficiency of miRNA in cell lines based on downregulation of *TWF1* (*PTK9*) mRNA following transfection with *miR-1* (This method was recommended by the manufacturer).

### 4.4. Cell Proliferation, Migration, and Invasion Assays

A498 and 786-O cells were transfected with 10 nM miRNAs or siRNAs by reverse transfection. Cell proliferation was determined by XTT assay using a Cell Proliferation Kit II (Sigma-Aldrich, St. Louis, MO, USA). Cell migration was evaluated with wound healing assays. Cell invasion was analyzed using modified Boyden chambers containing Transwell-precoated Matrigel membrane filter inserts. These assays were performed as described previously [45,46,47].

### 4.5. miRNA Incorporated into RISC by Ago2 Immunoprecipitation

To confirm that exogenous *miR-149-5p* or *miR-149-3p* were incorporated into RISC, we performed immunoprecipitation assays using a microRNA Isolation Kit, Human Ago2 (Wako, Osaka, Japan) as described previously [19]. The expression levels of miRNAs bound to Ago2 were measured by TaqMan RT-qPCR. The miRNA expression data were normalized to the expression of miR-26a (product ID: 000404; Applied Biosystems), which was not affected by *miR-149-5p* or *miR-149-3p* expression.

### 4.6. Selection of Putative Target Genes Regulated by miR-149-5p and miR-149-3p in RCC Cells

To identify target genes of *miR-149-5p* and *miR-149-3p* in ccRCC, we performed in silico analyses and genome-wide gene expression analyses. We used the TargetScanHuman database (Release 7.1; http://www.targetscan.org/vert_71/), TCGA database (https://cancergenome.nih.gov/), and OncoLnc (http://www.oncolnc.org/) for in silico analyses [20,21,22]. The genome-wide gene expression analyses were performed using microarray data and gene expression profiles (GEO database; accession number: GSE22541 and GSE36895). The microarray data were deposited into the GEO database (accession number: GSE100746). We merged these datasets and selected putative *miR-149-5p* and *miR-149-3p* target genes.

### 4.7. TCGA Database Analysis of ccRCC

To investigate the clinical significance of target genes, we used the TCGA database from OncoLnc and cBioPortal (http://www.cbioportal.org/) [20,21,22]. We analyzed Kaplan-Meier survival curves and log-rank tests for target genes, and evaluated the lower expression group and the higher expression group of *FOXM1* by T stage and metastatic cases.

### 4.8. Western Blot Analysis

Immunoblotting was conducted with monoclonal anti-FOXM1 antibodies (1:1000 dilution; #5436; Cell Signaling Technology, Danvers, MA, USA) and with anti-glyceraldehyde 3-phosphate dehydrogenase (GAPDH) antibodies (1:1000 dilution; ab8245; Abcam, Cambridge, UK) as a loading control. The procedures were performed as previously described [45,46,47].

### 4.9. Immunohistochemistry Using Tissue Microarrays

We used a tissue microarray of renal cell carcinoma samples obtained from US Biomax (Derwood, MD, USA; cat no. KD806), which contained a total of 80 renal tissues (clear cell carcinoma, *n* = 68; carcinoma sarcomatodes, *n* = 2; normal renal samples, *n* = 10). Detailed information on these samples is available at http://www.biomax.us/tissue-arrays/Kidney/KD806.

### 4.10. Statistical Analysis

The relationships between the two groups and expression values obtained by RT-PCR were analyzed using Mann–Whitney *u*-tests. The correlations between *miR-149-5p* and *miR-149-3p* expression were evaluated using Spearman’s rank test. The relationships among more than three variables and numerical values were analyzed using Bonferroni-adjusted Mann–Whitney *u*-tests. Survival analysis was carried out using the Kaplan–Meier method and log-rank tests with JMP software (version 12, SAS Institute Inc., Cary, NC, USA). Other analyses were performed using Expert StatView software (version 5.0, SAS Institute Inc., Cary, NC, USA) for these analyses.

## 5. Conclusions

Both strands of pre-*miR-149*, *miR-149-5p* (guide strand) and *miR-149-3p* (passenger strand), acted as antitumor miRNAs in RCC cells. *FOXM1* was regulated by these miRNAs, and an overexpression of *FOXM1* was observed in RCC clinical specimens. The aberrant expression of *FOXM1* enhanced cancer cell aggressiveness, and high expression of *FOXM1* was significantly associated with a poor prognosis of this disease. The identification of antitumor-mediated oncogenic networks may lead to a better understanding of RCC pathogenesis.

## Figures and Tables

**Table 1:** Characteristics of patients with clear cell renal cell carcinoma (ccRCC).

No.	Age	Sex	Pathology	Grade	pT	N	M	INF	v	ly	eg/ig	fc	im	rc	rp	s
1	71	F	clear cell	G2	T1a	0	0	a	0	0	eg	1	0	0	0	0
2	74	M	clear cell	G1 > G2	T1b	0	0	a	0	0	eg	1	0	0	0	0
3	59	M	clear cell	G3 > G2	T1b	0	0	a	0	0	eg	1	0	0	0	0
4	79	M	clear cell	G2 > G3 > G1	T1a	0	0	a	0	0	eg	1	0	0	0	0
5	52	M	clear cell	G2 > G3	T1b	0	0	a	0	0	eg	1	1	0	0	0
6	76	F	clear cell	G2 > G3	T3a	0	0	a	1	0	eg	1	0	0	0	0
7	64	M	clear cell	G2 > G3 > G1	T3a	0	1	b	1	0	ig	0	1	1	0	0
8	67	M	clear cell	G2 > G3 > G1	T3a	0	0	b	1	0	ig	1	0	0	0	0
9	59	M	clear cell	G3	T3a	0	0	b	1	0	ig	0	0	0	0	0
10	73	M	clear cell	G1 >> G3	T2a	0	0	a	0	1	eg	1	0	0	0	0
11	77	M	clear cell	G1 > G2	T1b	0	0	a	0	0	eg	1	0	0	0	0
12	51	F	clear cell	G2 > G1 > G3	T3b	0	0	b	1	0	ig	0	0	0	0	0
13	84	F	clear cell	G2	T1a	0	0	a	0	0	eg	0	0	0	0	0
14	78	M	clear cell	G2 > G1 >> G3	T1b	0	0	b	0	0	eg	1	0	0	0	0
15	44	M	clear cell	G2 > G1	T1a	0	0	b	0	0	eg	1	0	0	0	0
16	57	M	clear cell	G2	T1b	0	0	a	0	0	eg	0	0	0	0	0

INF: infiltration; v: vein; ly: lymph node; eg: expansive growth; ig: infiltrative growth; im: intrarenal metastasis; rc: renal capsule invasion; rp: pelvis invasion; s; sinus invasion.

**Table 2:** Putative target genes regulated by *miR-149-5p* and *miR-149-3p* in RCC cells.

Gene Symbol	Gene Name	Site Counts		Microarray (log2 Ratio)		GEO	TCGA
		*miR-149-5p*	*miR-149-3p*	*miR-149-5p*	*miR-149-3p*	Fold Change	*p* Value
*FOXM1*	forkhead box M1	1	1	−1.359	−0.834	1.533	1.5 × 10^−6^
*SIPA1*	signal-induced proliferation-associated 1	1	3	−2.022	−0.732	1.071	6.4 × 10^−6^
*CXCL5*	chemokine (C-X-C motif) ligand 5	1	1	−1.032	−0.650	1.961	2.39 × 10^−6^
*LMNB2*	lamin B2	1	4	−0.767	−0.909	1.438	3.67 × 10^−6^
*GBP2*	guanylate binding protein 2, interferon-inducible	1	4	−2.484	−1.278	1.664	8.57 × 10^−6^
*FBXL16*	F-box and leucine-rich repeat protein 16	2	4	−2.451	−0.685	2.203	0.00927 *
*BTNL9*	butyrophilin-like 9	1	3	−1.161	−0.519	1.255	0.0187 *
*APOBEC3C*	apolipoprotein B mRNA editing enzyme, catalytic polypeptide-like 3C	2	1	−1.086	−0.672	1.805	0.0424
*MARVELD1*	MARVEL domain containing 1	2	4	−0.902	−2.176	1.019	0.188
*IKZF1*	IKAROS family zinc finger 1 (Ikaros)	1	2	−1.133	−0.742	1.350	0.401
*TTYH3*	tweety family member 3	1	7	−1.933	−1.480	1.096	0.57
*SLC29A4*	solute carrier family 29 (equilibrative nucleoside transporter), member 4	1	11	−1.115	−0.868	2.936	0.62
*DDB2*	damage-specific DNA binding protein 2, 48kDa	2	1	−0.760	−1.295	2.209	0.757
*PHKA2*	phosphorylase kinase, alpha 2 (liver)	2	3	−0.550	−1.281	1.872	0.943

* Poor prognosis with low expression.
